# Correlation Between the Type of Acute Coronary Syndrome With the Needs of Hospitalized Patients

**DOI:** 10.5539/gjhs.v8n7p126

**Published:** 2015-11-18

**Authors:** Maria Polikandrioti, John Goudevenos, Lampros K. Michalis, Ioannis Koutelekos, Elpida Georgiadi, Kostas Karakostas, Moses Elisaf

**Affiliations:** 1 Nursing Department, Technological Institute of Athens, Greece; 2 Cardiology Department, Ioannina University Hospital, Greece; 3 Nurse Thriasio Hospital, Athens, Greece; 4 Department of Internal Medicine, University of Ioannina, Greece

**Keywords:** Acute Coronary Syndromes, needs of hospitalized patients

## Abstract

**Introduction:**

Acute Coronary Syndromes (ACS) comprise life-threatening health problems that demand emergency care and immediate intervention. As patients are abruptly transitioning from healthy state into suffering, they consequently experience several needs, mainly attributed to the type of the syndrome including the therapeutic regimen.

**Objectives:**

To access the correlation between the type of acute coronary syndrome (ACS) with the needs of hospitalized patients.

**Methods:**

A sample of 454 hospitalized patients with ACS, recruited from 4 hospitals in Greece, was enrolled in the study. Data were collected by the completion of questionnaire which apart from socio-demographic and clinical characteristics, it also included the questionnaire “Needs of hospitalized patients with coronary artery disease” which is consisted 6 subscales: a) need for support and guidance, b) need for information from the medical-nursing staff, c) need for being in contact with other patient groups and ensuring communication with relatives, d) need for individualized treatment and for the patient’s personal participation to his/her treatment e) need to meet the emotional and physical needs f) need to trust the medical-nursing staff. Statistical methods used were Kolmogorov-Smirnov test, chi2 test of independence, Kruskal wallis-test and multiple regression.

**Results:**

The type of ACS was statistically significant correlated with the place of residence (p=0.002), management of disease (p<0.001) and prior experience of hospitalization (p=0.003). All six needs were statistically significantly correlated with the type of ACS, (p<0.001 for the need for support and guidance, p<0.001 for the need to be informed from the medical and nursing staff, p<0.001 for the need for being in contact with other patient groups, and ensuring communication with relatives, p<0.001 for the need for individualized treatment and for the patient’s personal participation to his/her treatment, p<0.001 for the need to meet the emotional needs and physical needs and p=0.010 for the need to trust the medical and nursing staff). More specifically, patients with angina considered all six needs to be less significant than patients with unstable angina and myocardial infarction. These results were confirmed by the multiple linear regression after controlling for potential confounders.

**Conclusions:**

Needs of hospitalized patients should be assessed in daily clinical practice according to the type of the syndrome.

## 1. Introduction

Acute coronary syndromes impose sudden changes in patients’ lives immediately after diagnosis. For example, patients with acute myocardial infarction are abruptly transitioning from healthy state into suffering, that requires adaption to the disease and the new way of living including modification of risk factors (Polikandrioti & Νtokou, 2011; Asadi-Lari, Packham, & Gray, 2003a; Asadi-Lari, Packham, & Gray, 2003b).

Interestingly enough, assessing needs of hospitalized patients with acute coronary syndromes has beneficial effect on disease management. For instance, symptoms of coronary artery disease are sometimes so threatening to patients’ lives that they feel unable to handle them due to the lack of knowledge. Equally important is the deep understanding of modifiable risk factors to prevent an oncoming episode of coronary syndrome. Therefore, patients need guidance, support and elaborate information to promote health-related behavior change. (Polikandrioti & Νtokou, 2011; Asadi-Lari, Packham, & Gray, 2003a; Asadi-Lari, Packham, & Gray, 2003b).

In daily clinical practice, the main advantage of needs’ assessment is individualized care planning. Significantly more, this approach enhances the power in doctor-patient relation, thus facilitating the long-term treatment success and patient’s adjustment to illness. Indeed, nowadays the disease management is not only limited to the improvement of clinical state but also focuses on quality of care including patients’ empowerment to retain prior roles and activities. Ultimately, needs’ assessment may indicate potential areas that merit further evaluation such as the gap between patients’ health needs and availability of health care services (Asadi-Lari, Packham, & Gray, 2003c; James, 1999; Stevens, & Gillam, 1998; Wilkinson, & Murray, 1998; Hawe, 1996).

The **aim** of the present study was to explore the correlation between the type of acute coronary syndrome (ACS) with the needs of hospitalized patients.

## 2. Method and Material

### 2.1 Study Population

In the present study participated 454 hospitalized patients of four hospitals in Attica with acute coronary syndrome: a) angina b) unstable angina (UA) and c) myocardial infarction.

The hospitals enrolled in the present study were similar and more in detail, they were all ’General public hospitals’ admitting patients of acute coronary syndrome at their Emergency Department.

Data were collected from February 2014 to December 2014.

The present sample was a convenience sample. The inclusion criteria for the research were: a) good knowledge, apprehension and use of the Greek language b) having being hospitalized for at least 3 days and c) being diagnosed with acute coronary syndrome.

Patients who met the entry criteria and volunteered in the present study were informed for the purposes of this study, by the researchers. Then, the researchers asked for the patients’ written consent for participation. Only individuals who gave their consent were included in the study.

### 2.2 Instrument

Data collection was performed by the method of interview by the researchers who developed this questionnaire to fully serve the purposes of the study. More in detail, the questionnaire was comprised of : a) demographic and clinical characteristics: Gender, age, marital and educational status, place of residence, number of children, years with heart failure, prior experience of hospitalization and the degree of information and b) the questionnaire ‘Needs of hospitalized patients with coronary artery disease’ by Polikandrioti et al. (2011) which included 39 questions regarding needs of patients with coronary artery disease during hospitalization.

The questionnaire ‘Needs of hospitalized patients with coronary artery disease’ consisted of 6 subscales of needs : a) need for support and guidance, b) need for information from the medical-nursing staff, c) need for being in contact with other patient groups, and ensuring communication with relatives, d) need for individualized treatment and for the patient’s personal participation to his/her treatment e) need to meet the emotional needs (e.g anxiety, fear, loneliness) and the physical needs (such as relaxation, sleep, better conditions during hospitalization), and f) need to trust the medical-nursing staff.

Participants had to report how significant was each question for them. The Likert type four-scale was used to answer all questions. The four different scales were represented the following answers: No, Little, Μuch and Too Much. Answers for each subscale of needs were summed up, leading to six different scores. Lower score means low level of need.

This questionnaire was constructed a) according to the Kristjandottir (1995) questionnaire, that evaluates the needs of hospitalized children’s parents and later on had been translated and used in Greece by Kyritsi et al. (2005) and b) after evaluating information about patients’ needs from literature.

The reliability and the validity of the questionnaire ‘Needs of hospitalized patients with coronary artery disease’ had been tested in previous research by Polikandrioti et al. (2011) in Greek population.

It took between 15 and 30 minutes to complete the questionnaire.

The study was approved by the Medical Research Ethics Committee of each hospital and was conducted in accordance to the World Medical Correlation’s Declaration at Helsinki (1989).

### 2.3 Statistical Analysis

Kolmogorov-Smirnov test was used to assess the normality of continues variables (i.e. patients’ needs etc). Categorical variables are summarized as absolute and relative (%) frequencies, while continuous variables as mean ± standard deviation and as median (interquartile range) when they are normally distributed or skewed, respectively. Correlations between patients’ characteristics and the type of acute coronary syndrome (ACS) were tested using chi2 test of independence. For the correlation between the length of stay in hospital, the needs and the type of ACS, Kruskal wallis-test was performed with bonferroni correction for multiple comparisons.

Finally, multiple regression was performed in order to evaluate the correlation between patients’ needs and type of ACS after adjusting for possible confounders (factors that were found to be significantly correlated with the needs at a univariate basis). The results are presented as β coefficients and 95% confidence interval (95% CI).

All reported P values were based on two-sided hypotheses and compared to a significant level of 5%. All statistical analysis was carried out using SPSS program, version 20 (SPSS Inc, Chicago, Il, USA).

## 3. Results

### 3.1 Correlation of Demographics and Type of ACS

Table 1 presents the correlation of patients’ characteristics and the type of ACS. The type of ACS was statistically significant correlated with the place of residence (p=0.002), management of disease (p<0.001), prior experience of hospitalization (p=0.003) and length of stay in hospital (p<0.001).

**Table 1:** Correlation between socio-demographic, clinical characteristics of patients and type of heart disease

Socio-Demographics	UA N(%)	Myocardial Infarction N(%)	Angina N(%)	p-Value
Gender				
*Male*	74 (23.49%)	160 (50.79%)	81 (25.71%)	0.809
*Female*	35 (25.18%)	66 (47.48%)	38 (27.34%)	
Age (Years)				
*<50*	14 (24.14%)	32 (55.17%)	12 (20.69%)	0.950
*51-60*	28 (23.14%)	61 (50.41%)	32 (26.45%)	
*61-70*	33 (23.91%)	69 (50.00%)	36 (26.09%)	
*>70*	34 (24.82%)	64 (46.72%)	39 (28.47%)	
Marital Status				
*Married/Living Together*	78 (23.64%)	169 (51.21%)	83 (25.15%)	0.581
*Single/Divorced/Separated/Widowed*	31 (25.00%)	57 (45.97%)	36 (29.03%)	
Educational Status				
*Primary Education*	48 (21.05%)	116 (50.88%)	64 (28.07%)	0.344
*Secondary Education*	45 (26.95%)	77 (46.11%)	45 (26.95%)	
*University/Master-Phd*	15 (26.32%)	32 (56.14%)	10 (17.54%)	
Place Of Residence				
*Attica*	36 (17.06%)	103 (48.82%)	72 (34.12%)	**0.002**
*Capital City*	29 (31.87%)	50 (54.95%)	12 (13.19%)	
*Small Town*	20 (28.17%)	32 (45.07%)	19 (26.76%)	
*Rural*	24 (29.63%)	41 (50.62%)	16 (19.75%)	
Number Of Children				
*None*	15 (30.61%)	23 (46.94%)	11 (22.45%)	0.791
*One*	14 (20.59%)	35 (51.47%)	19 (27.94%)	
*Two*	58 (25.66%)	109 (48.23%)	59 (26.11%)	
*Three Or More*	22 (19.82%)	59 (53.15%)	30 (27.03%)	
Management Of Disease				
*Conservative*	92 (36.65%)	83 (33.07%)	76 (30.28%)	**<0.001**
*Intervention (PCI /CABG/Pacemaker)*	17 (10.56%)	101 (62.73%)	43 (26.71%)	
Degree Of Information				
*Much*	32 (19.39%)	83 (50.30%)	50 (30.30%)	0.137
*Enough*	54 (24.77%)	114 (52.29%)	50 (22.94%)	
*Little/Not At All*	23 (32.39%)	29 (40.85%)	19 (26.76%)	
Years With Problem				
*<1*	59 (24.69%)	129 (53.97%)	51 (21.34%)	0.119
*2-5*	34 (25.37%)	59 (44.03%)	41 (30.60%)	
*>5*	16 (19.75%)	38 (46.91%)	27 (33.33%)	
Prior Experience Of Hospitalization Due To Problem				
*Yes*	44 (20.85%)	96 (45.50%)	71 (33.65%)	**0.003**
*No*	65 (26.75%)	130 (53.50%)	48 (19.75%)	
	**Median(IQR)**	**Median(IQR)**	**Median(IQR)**	
Length Of Stay In-Hospital	4 (4-5)*	5 (4-6)	5 (4-5)	**<0.001**

* statistically significant different score from myocardial infraction group, after bonferroni correction (multiple comparisons).

More specific, higher percentages of UA and myocardial infarction were observed in capital cities (31.87% and 54.95% respectively) but higher percentages of angina were observed in Attica (34.12%).

Patients with UA were more likely to follow conservative therapy (36.65%) and patients with myocardial infarction were more likely to follow interventions (62.73%).

Furthermore, patients with myocardial infarction and angina were more likely to have prior experience to hospitalization (45.5% and 33.65% respectively) than patients with UA (20.85%).

Lastly, patients with myocardial infarction (median 5) stayed in hospital longer than the other patients (medians 4 and 5).

### 3.2 Patients’ Needs

Table 2 presents how patients evaluate the level of significance of their needs. Patients with ACS considered all six needs of low significance, once the median scores of each need were close to the lower limit of the needs ranges.

**Table 2:** Descriptive data of the sub-scales assessing the importance of the needs of patients

Patients’ needs (range)	Median (IQR)
*Need for support and guidance (9-36)*	11(9-15)
*Need to be informed from the medical and nursing staff (8-32)*	9(8-12)
Need for being in contact with other patient groups, and ensuring communication with relatives *(6-24)*	12(7-15)
*Need for individualized treatment and for the patient’s personal participation to his/her treatment (6-24)*	8(6-11)
*Need to meet the emotional needs (eg, anxiety, fear, loneliness) and physical needs (such as relaxation, sleep, better conditions of treatment) (7-28)*	9(7-12)
*Need to trust the medical and nursing staff (2-8)*	2(2-2)

### 3.3 Correlation of Needs and Type of Heart Problems

All six needs were statistically significantly correlated with the type of ACS with a p-value <0.001 and p=0.010, Table 3. More specifically, patients with angina considered all six needs to be less significant than patients with UA and myocardial infarction, especially after multiple comparisons between the three groups even for the need “to trust the medical and nursing staff” where the three medians coincide. Moreover, patients with myocardial infarction considered the need for communication, the need for individualization of care and the need to meet the emotional and physical needs statistically less significant than patients with UA.

**Table 3:** Correlation between type of heart disease and needs

Needs	UA Median(IQR)	Myocardial Infarction Median(IQR)	Angina Median(IQR)	p-value
*Need for support and guidance (9-36)*	12(9-15)	12(9-16)	9(9-11)*	**<0.001**
*Need to be informed from the medical and nursing staff (8-32)*	10(8-13)	9(8-12)	8(8-10)*	**<0.001**
*Need* for being in contact with other patient groups, and ensuring communication with relatives *(6-24)*	14(9.5-17)	12.5(8-15)**	8(6-12)*	**<0.001**
*Need for individualized treatment and for the patient’s personal participation to his/her treatment (6-24)*	10(8-13)	9(6-11)**	6(6-9)*	**<0.001**
*Need to meet the emotional needs (eg, anxiety, fear, loneliness) and physical needs (such as relaxation, sleep, better conditions of treatment) (7-28)*	12(9-13)	9(7-13)**	7(7-9)*	**<0.001**
*Need to trust the medical and nursing staff (2-8)*	2(2-2)	2(2-2)	2(2-2)**	**0.010**

* statistically significant different score compared to other 2 groups after bonferonni correction, multiple comparisons (p<0.05).

** statistically significant different score compared to other UA group after bonferonni correction, multiple omparisons (p<0.05).

Linear multiple regression (Table 4) revealed that patients with angina considered the need for support and guidance 2.67 points less significant than patients with UA. Also patients with angina considered the need to be informed 1.33 points less significant, the need for communication 3.69 points less significant, the need for individualization of care 2.79 points less significant, the need to meet their needs 3.24 points less significant, and the need to trust the staff 0.3 points less significant than patients with UA problems, after adjusted for possible confounders (characteristics that are statistically significant correlated with the needs).

**Table 4:** Multiple regression for the correlation between type of heart disease and needs (adjusted for basic characteristics)

Sub-scale	Heart Problem	β coefficient	p-value
***Need for support and guidance***	**UA**	Ref. Cat.	
	**Myocardial Infarction**	-0.22(-1.10,0.66)	0.631
	**Angina**	-2.67(-3.67,-1.67)	**<0.001**

Need to be informed from the medical and nursing staff*	**UA**	Ref. Cat.	
	**Myocardial Infarction**	-0.36(-1.12,0.39)	0.348
	**Angina**	-1.33(-2.20,-0.46)	**0.003**

Need for being in contact with other patient groups, and ensuring communication with relatives**	**UA**	Ref. Cat.	
	**Myocardial Infarction**	-0.99(-1.97,-0.01)	**0.048**
	**Angina**	-3.69(-4.73,-2.64)	**<0.001**

***Need for individualized treatment and for the patient’s personal participation to his/her treatment***	**UA**	Ref. Cat.	
	**Myocardial Infarction**	-1.10(-1.85,-0.35)	**0.004**
	**Angina**	-2.79(-3.63,-1.94)	**<0.001**

Need to meet the emotional and physical needs***	**UA**	Ref. Cat.	
	**Myocardial Infarction**	-1.29(-1.98,-0.59)	**<0.001**
	**Angina**	-3.24(-4.04,-2.44)	**<0.001**

Need to trust the medical and nursing staff****	**UA**	Ref. Cat.	
	**Myocardial Infarction**	-0.09(-0.27,0.09)	0.310
	**Angina**	-0.30(-0.49.-0.10)	**0.004**

* adjusted for marital status, degree of information, length of stay.

** adjusted for place of residence, management of disease, years of problem, length of stay.

*** adjusted for place of residence, years of problem.

**** adjusted for degree of information.

Furthermore patients with myocardial infarction considered the need for communication 0.99 points less significant, the need for individualization of care 1.10 points less significant and the need to meet their needs 1.29 points less significant than patients with UA problems.

## 4. Discussion

Needs’ assessment based on the type of coronary syndrome represents a new area of interest to health professionals who provide care in daily cardiac clinical practice.

The results of the present study showed that the type of ACS was correlated with the place of residence, management of disease, prior experience of hospitalization and length of hospital stay.

Participants with UA were more likely to follow conservative therapy and those with myocardial infarction were more likely to follow intervention therapy.

Management of the disease varies according to the type of the syndrome. More in detail, unstable angina requires monitoring for some days in order to exclude the possibility of a myocardial infarction whereas the treatment choice in patients with myocardial infarction is coronary intervention or thrombolytic therapy (Sami & Willerson, 2010; Asadi-Lari, Packham, & Gray, 2003a).

The finding that patients with myocardial infarction were more likely to have longer hospital stay, is mainly attributed to the need for closer monitoring and prevention of further myocardial damage during hospitalization.

Moreover, patients with myocardial infarction were more likely to have prior experience to hospitalization, an issue that is not rare since according to Shen et al. (2014) 20% of patients with acute myocardial infarction had a history of a prior event.

Failure of patients’ participation in cardiac rehabilitation programs as well as non compliance to the therapeutic regimen may increase the possibility of a new cardiac event or a re-hospitalization (Smith, Negrelli, Manek, Hawes, & Viera, 2015; Sai & Willerson, 2010).

Research by Tisminetzky et al. (2015) found that readmission of hospitalized patients for an initial NSTEMI myocardial infarction within 30 days was more frequent in patients with prolonged index hospitalization. According to Wang et al. (2012) factors that may decrease hospitalization are: effective management of cardiovascular risk factors, the therapeutic regimen as well as accurate diagnosis mainly attributed to the use of more sensitive troponin biomarker assays. Socioeconomic and marital status, level of education and social network are according to Damiani et al. (2015) the main predictors of readmissions in elderly patients with acute myocardial infarction.

Data showed that higher percentages of UA and myocardial infarction were observed in capital cities whereas higher percentages of angina were observed in Attica, thus raising significant concerns about the relation between the place of residence and acute coronary syndromes. In support of this view there is evidence that birth place is related with life style risk factors or atherosclerosis (Tyynela et al., 2010; Tyynela et al., 2009).

All six needs were statistically significantly correlated with the type of ACS.

To the best of our knowledge, there is not a study evaluating patients’ needs according to the type of acute coronary syndrome. However, Asadi-Lari et al. (2005) developed a tool for exploring cardiac patients’ needs (Nottingham Health Needs Assessment; NHNA) which included different dimension of needs compared to the present study. The same researchers in a prior study showed that 242 patients admitted to Acute Cardiac Unit were in need of more social and physical support. Specifically more, the researchers put emphasis on two categories of needs: a) general health care needs according to socio-demographic characteristics of cardiac patients and b) specific needs unique to each patient group. The second approach is, up to some extent, similar to the present study because partially it supports that patients’ needs vary according to the type of acute coronary syndrome. Finally and most strikingly, the same researchers stated that unmet needs of cardiac patients are related with increased morbidity and mortality (Asadi-Lari, Packham, & Gray, 2003a). On the basis of this thought, we sought to determine cardiac patients’ needs.

Analysis also revealed that patients with angina considered all six needs to be less significant than patients with UA and myocardial infarction. Furthermore, patients with myocardial infarction considered the need for communication, the need for individualization of care and the need to meet the emotional and physical needs statistically less significant than patients with UA.

A possible explanation is that patients are more concerned about the nature of the disease, thus reporting these needs as less significant. An alternative suggestion is that this finding reflects the way that patients perceive their health state. However, a second assessment of needs at hospital discharge may be beneficial for the reason that patients start to consider the impact of the disease on their lives when they are about to return home.

Interestingly, the research by Kilonzo and O’Connell (2011) supported that patients after coronary intervention pay more attention to the outcome of the disease and their relation with nurses. Researches by Timmins (2005), Timmins and Kaliszer (2003) showed that hospitalized patients with acute coronary syndrome are more focused on survival issues, such as addressing the symptoms rather than issues related to lifestyle changes. Research by Scot and Thompson (2003) claimed that post-myocardial infarction patients are more concerned about modifiable risk factors, followed by information on anatomy, therapy and physical activity.

In regard to the way patients perceive the disease, the research by Lauck et al. (2009) showed that patients who underwent angioplasty usually underestimate the significance of the disease for various reasons such as the short time of procedure and hospital stay, the immediate improvement of symptoms and return to prior activities. According to Campbell and Torrance (2005) approximately 42% of patients undergoing angioplasty believed that they no longer suffered from coronary disease.

Similarly, in the study conducted by Gentz (2000) patients reported coronary angioplasty as a routine intervention and considered significant the need for information. In the research by Kattainen et al. (2004) patients undergoing Coronary Artery Bypass Graft Surgery (CABG) reported as of high importnace the need of information regarding their psychosocial functioning both preoperatively and postoperatively and in the rehabilitation period.

Though several theories may be suggested in an effort to explain why patients of each type of acute coronary syndrome consider the needs less or more significant, it is strongly recommended that needs’ assessment can be a valuable tool for health professionals in cardiac clinical setting.

### 4.1 Limitations of the Study

The sample of the present study was a convenience one. Consequently it is not representative of patients with acute coronary syndrome in Greece, thus limiting the ability of results’ generalization.

## 5. Conclusions

All six needs were statistically significantly correlated with the type of ACS, (the need for support and guidance, the need to be informed from the medical and nursing staff, the need for being in contact with other patient groups, and ensuring communication with relatives, the need for individualized treatment and for the patient’s personal participation to his/her treatment, the need to meet the emotional needs and physical needs and the need to trust the medical and nursing staff).

Planning individualized holistic care should incorporate evaluation of patients’ needs according to the nature of the syndrome.

Needs’ assessment is an essential step in facilitating recovery of acute coronary syndrome survivors.
